# Energetic savings of bow-riding dolphins

**DOI:** 10.1038/s41598-024-81920-y

**Published:** 2024-12-03

**Authors:** Lorenzo Fiori, Randall W. Davis, Bernd Würsig, Dara N. Orbach

**Affiliations:** 1 Department of Life Sciences, Texas A&M University-Corpus Christi, Corpus Christi, TX USA; 2 Azores Delphis Project, Ponta Delgada, Azores Portugal; 3 Department of Marine Biology, Texas A&M University at Galveston, Galveston, TX USA

**Keywords:** Anthropogenic interaction, Bow-riding, Dusky dolphins, Locomotion, Respiration, Unoccupied aerial system, Ecology, Ecology

## Abstract

Bow-riding occurs when dolphins swim in the pressure waves at the front of a vessel. Bow-riding is hypothesized to be “fun” for dolphins or to save them energy although the energetics have not been explored. An UAS (Unoccupied Aerial System) was used to follow and video-record adult dusky dolphins (*Lagenorhynchus obscurus*) bow-riding in front of a research vessel or free-swimming off Kaikoura, New Zealand. Videos of individual dolphins swimming in a linear direction at consistent speeds were analyzed with respiration rate used as a proxy for energy expenditure (bow riding *n* = 51; free-swimming *n* = 62). The respiration rates of bow-riding dolphins remained relatively constant across swimming speeds and were 45% lower than free-swimming dolphins at speeds exceeding 4 m/s, indicating substantial energetic savings. The respiration rates of free-swimming dolphins increased exponentially with speed, suggesting that dolphins incur comparatively high energetic expenditures from swimming rapidly. This research advances understanding of the biological function of bow-riding behavior and supports the energy saving hypothesis. Swimming energetics can be used to assess the impacts of anthropogenic disturbances to dolphin energy budgets.

## Introduction

Dolphins frequently bow-ride in front of advancing vessels and may benefit from the lift generated by the hull moving through the water^1^. Similarly, dolphins ride in the wakes of advancing vessels^2,3^. Bow- and wake-riding on vessels follow similar principles to riding on waves produced by the ocean, other large cetaceans, or sharks^4–6^. Once positioned in the pressure wave, dolphins rarely need to strike their tails to thrust forward^7^ except to breathe^2^. Wake-riding behavior may be learned as a neonate or calf by maintaining an echelon position next to the offspring’s mother, which provides the locomotion advantage of reduced drag forces; bottlenose dolphin (*Tursiops truncatus*) calves in the echelon-position reduced tail stroke amplitude by 22% and increased the distance covered per stroke by 19% compared to free-swimming calves^8^. Dolphins swimming in tight formation may save energy by riding on the wake produced by conspecifics^7^; for example, killer whales (*Orcinus orca*) swimming in the wake of conspecifics decreased their predicted tail beat frequency by 10% compared to the lead animal generating the wake^9^.

The biological function of dolphin bow-riding behavior has been debated for decades. Dolphins may bow-ride to efficiently travel from one location to another^2^; bottlenose dolphins have been observed riding on the bows of large ships for more than 20 km^6^. As after bow-riding dolphins frequently return to the location where they first encountered vessels, the behavior may not function for travel in all instances. Bow-riding may be a play behavior^10^ as dolphins spend energy to approach vessels and whales to bow-ride akin to humans who spend energy paddling to ride a wave^11^. Bow-riding may have long-term negative effects on dolphins if it causes a reduction of the time spent foraging and/or resting^12,13^.

It has been challenging to quantify the energetic advantage to locomotion associated with bow-riding or wake riding, especially of untrained free-swimming dolphins. Research on cetacean energetic costs of swimming has been limited to a few dolphin species under human care or telemetry tags on large species in the wild^14–17^. Because tagging is invasive and generally not conducive for small and fast swimming dolphins^18^, proxies are needed to measure energetic costs for free-swimming dolphins. Respiration rate has been used as a proxy of energy spent during locomotion among dolphins engaged in near-surface exercise^15^. The tidal volume of a dolphin (quantity of air expelled and inhaled for each breath) is approximately the maximum air volume that can be expelled after the deepest possible breath^15,17^. Therefore, dolphins can only increase the quantity of air exchanged during exercise by increasing their respiration rate^15^.

Trained bottlenose dolphins (*Tursiops truncatus*) travelling at 3.8 m/s had a respiration rate of 5.5 breaths/min while wake-riding compared to 8.8 breaths/min while free-swimming^2^; the post-exercise lactate concentration was three times higher for the free-swimming dolphin, indicating substantial physiological advantages of wake-riding^2^. Respiration rate measurements of several dolphins swimming at different speeds are necessary to generalize conclusions about the energetic savings.

Tracking individual dolphins from a vessel to measure respiration rates is challenging as small oceanic dolphins can live in large aggregations of hundreds of individuals^19^. Small vertical take-off and landing (VTOL) unoccupied aerial systems (UAS) are a non-invasive tool^20^ increasingly used to conduct focal individual follows of small oceanic dolphins and porpoises^21–23^. VTOL UAS can be highly maneuverable and applied to collect focal individual behavioral data from fast swimming dolphins for extended periods^24^.

The objective of the study is to measure and compare the energetic costs of wild dolphin bow-riding and free-swimming behaviors at a range of speeds. Bow-riding dolphins are predicted to have lower respiration rates than free-swimming dolphins.

## Methods

### Study system

Dusky dolphins (*Lagenorhynchus obscurus*) are a small oceanic dolphin species occurring in the temperate and subpolar waters of the Southern Hemisphere. Dusky dolphins are distributed off the coasts of South America (Peru, Chile, Argentina), Southwestern Africa (Namibia, South Africa), New Zealand, and in proximity of the offshore islands of the Southern Indian and Atlantic Oceans^25^. Off Kaikoura, New Zealand (42^o^24’ S, 173° 41’ E), about 2,000 dusky dolphins are present at any time out of a national population estimate of 10,000 individuals^26^. South Bay, Kaikoura, is an open-ocean embayment of ~ 100 km², ranging from the Kaikoura peninsula in the North to the Haumuri Bluffs in the South. The Kaikoura Canyon is 1,200 m deep and extends from 500 m from the South Bay coastline to 60 km offshore^27^. Dusky dolphins opportunistically forage at night on the deep scattering layer of mesopelagic prey in the offshore section of the Kaikoura Canyon^28^. Dolphins return inshore to South Bay at dawn where they spend the day predominantly resting and socializing close to the coast, and begin returning offshore to the Kaikoura Canyon in the late afternoon^26^. Dusky dolphins swim in subgroups while moving inshore and offshore, which provided the opportunity to track individuals travelling at a range of speeds using a UAS.

### Survey design

Non-systematic surveys were conducted aboard a 6 m rigid-hull inflatable boat (RHIB, deep v-shaped mono hull, internal length = 4.44 m, internal beam = 1.54 m) with a single 80 hp 4-stroke outboard engine (full load hull displacement ~ 1100 kg, planing speed ~ 4 m/s) during December 2022. Research was conducted under permit #87,725-MAR issued by New Zealand’s Department of Conservation. The UAS operator held a Remote Pilot Certificate (#839465) issued by the Australian Civil Aviation Safety Authority. Surveys were conducted in good weather conditions (Beaufort Sea State < 3, wind speed < 15 knots) and involved a skipper, a UAS operator, and a trained observer. Data were collected opportunistically when dolphins travelled during the diel migration to/from the offshore foraging grounds and the coastal waters of South Bay. Survey occurred in the morning (from dawn to 11:00 am) and late afternoon (3:00 pm to sunset) to maximize the encounter rate with travelling dolphins and minimize glare on the water surface in the UAS videos. No data were collected during the voluntary code of conduct period from 11:30 am to 1:30 pm when dusky dolphins rest^29^. Dolphins engaged in socializing, resting, and milling behavioral states were not approached. Data on bow-riding were collected opportunistically when dolphins positioned themselves in the pressure wave in front of the research vessel. Vessel speed and direction were maintained constant until dolphins ceased bow-riding.

A four-propeller UAS (DJI Phantom 4 Pro v2; SZ DJI Technology Co., Ltd., Shenzhen, China) equipped with camera and polarized lens filter was used to record focal individual follow videos of bow-riding and free-swimming dusky dolphins. The UAS was hand-launched and retrieved from the research vessel. UAS operations were conducted in visual conditions (no rain, no proximate clouds, sky visibility > 10 km). During the flights, visual line of sight (VLOS) on the UAS was maintained by crew. The flight altitude was 20 m above sea level (ASL). Videos were recorded continuously at 60 frames per seconds (fps) during each flight for a maximum of 18 min. The video resolution was 4 K, 3840 × 2160 pixels. At the beginning of each focal individual follow, the UAS operator positioned the aircraft directly above the focal dolphin and communicated the video time (i.e., start time) to the observer. The follow continued until the focal dolphin could not be tracked continuously (e.g., animal dove, moved out of the video frame). At end of each focal individual follow, the UAS operator communicated the video time (i.e., end time) to the observer. Multiple follows of different dolphins were conducted per flight. Focal follow start and end times were used to facilitate the post-hoc selection of usable video segments.

### Video analysis

**Fig. 1:**
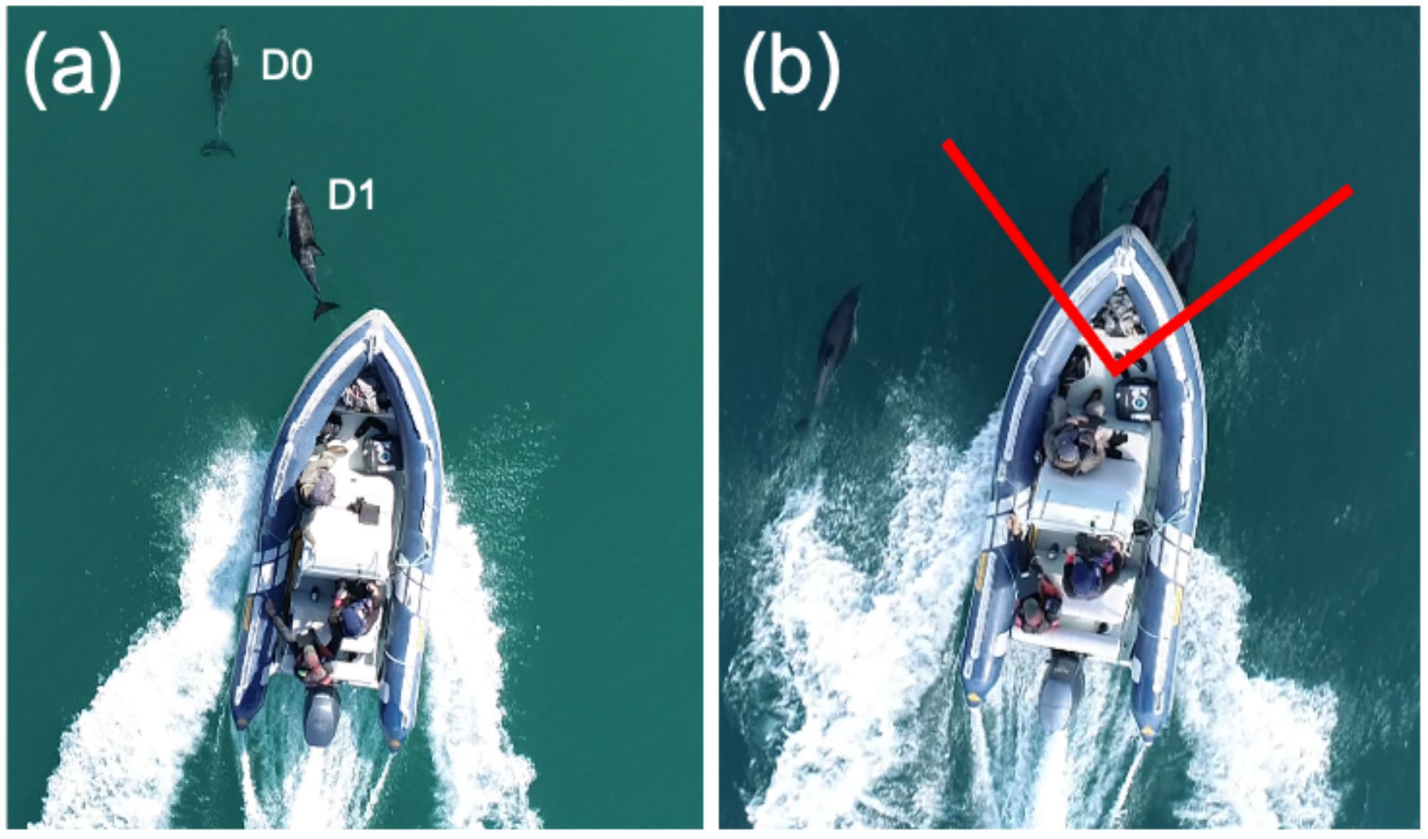
(**a**) Dolphins in bow-riding (D1) and not bow-riding (D0) positions. (**b**) The red angled line denotes the 90° area in front of the vessel where dolphins were considered bow-riding.

Videos were analyzed with VLC media player (videoland.org) at one-third speed. Segments of videos were selected for analysis when focal dolphins traveled in linear paths without rapid directional changes. Dolphins were categorized as bow-riding when positioned less than one body length distance from the bow of the vessel (Fig. 1a) and inside the 90° area in front of the bow (Fig. 1b). The number of dolphins bow-riding simultaneously was noted. When possible, multiple individuals were analyzed from the same bow-riding video segment. Free-swimming dolphins potentially influenced by the wake of the research vessel or engaged in circling and/or milling (meandering) behavior were excluded from the analysis. Each video segment analyzed consisted of the focal dolphin’s first through final recorded breath (opening of the blowhole) and terminated when the dolphin swam non-linearly, moved away from the bow-riding position, or moved out of the video frame and could be confused with a different dolphin (Fig. 1). Global positioning system (GPS) coordinates were obtained from the UAS flight metadata and visualized as subtitles in VLC media player. A distance calculator (omnicalculator.com/other/latitude-longitude-distance) was used to determine the distance traversed by each focal dolphin using initial and final video segment coordinates. The display times on the VLC media player were used to calculate the duration of each usable video segment. Swimming speed (m/s) was calculated as the distance (m) traveled divided by the duration of the video segment. The respiration rate (breaths/min) was calculated as the number of breaths divided by the duration of the video segment.

### Statistical analyses

Statistical analyses were conducted using SPSS Statistic 29 software (IBM, Armonk, NY, USA. 2022). Shapiro-Wilk and Levene’s tests were used to assess the assumptions of normality and homogeneity of variance. A one-tailed t-test for independent samples (not assuming equal variances) was used to compare the mean respiration rates of bow-riding and free-swimming dusky dolphins when traveling at the same speeds. A two-tailed t-test (assuming equal variances) was used to compare the mean respiration rates of dolphins bow-riding individually and with conspecifics. Speed distributions were compared with a two-tailed t-test for independent samples (assuming equal variances).

## Results

Thirteen surveys were conducted during December 2022, primarily in the morning (morning *n* = 12; afternoon *n* = 1) and in Beaufort Sea State 0–1. Thirteen hours of aerial videos were recorded. A total of 113 video segments of individual dusky dolphins travelling a linear path were selected and analyzed (bow-riding *n* = 51; free-swimming *n* = 62). Up to eight dolphins were observed bow-riding simultaneously (mean ± SE = 2.4 ± 0.2). The mean (± SE) duration of the video segments was 72 ± 5.9 s. Dolphins covered a mean distance (± SE) of 101 ± 5.6 m and swam at speeds ranging from 0.54 to 6.92 m/s. Based on the UAS GPS accuracy (± 1.5 m), a potential error of ± 1.5% was calculated for the mean distance and swimming speed.

**Fig. 2:**
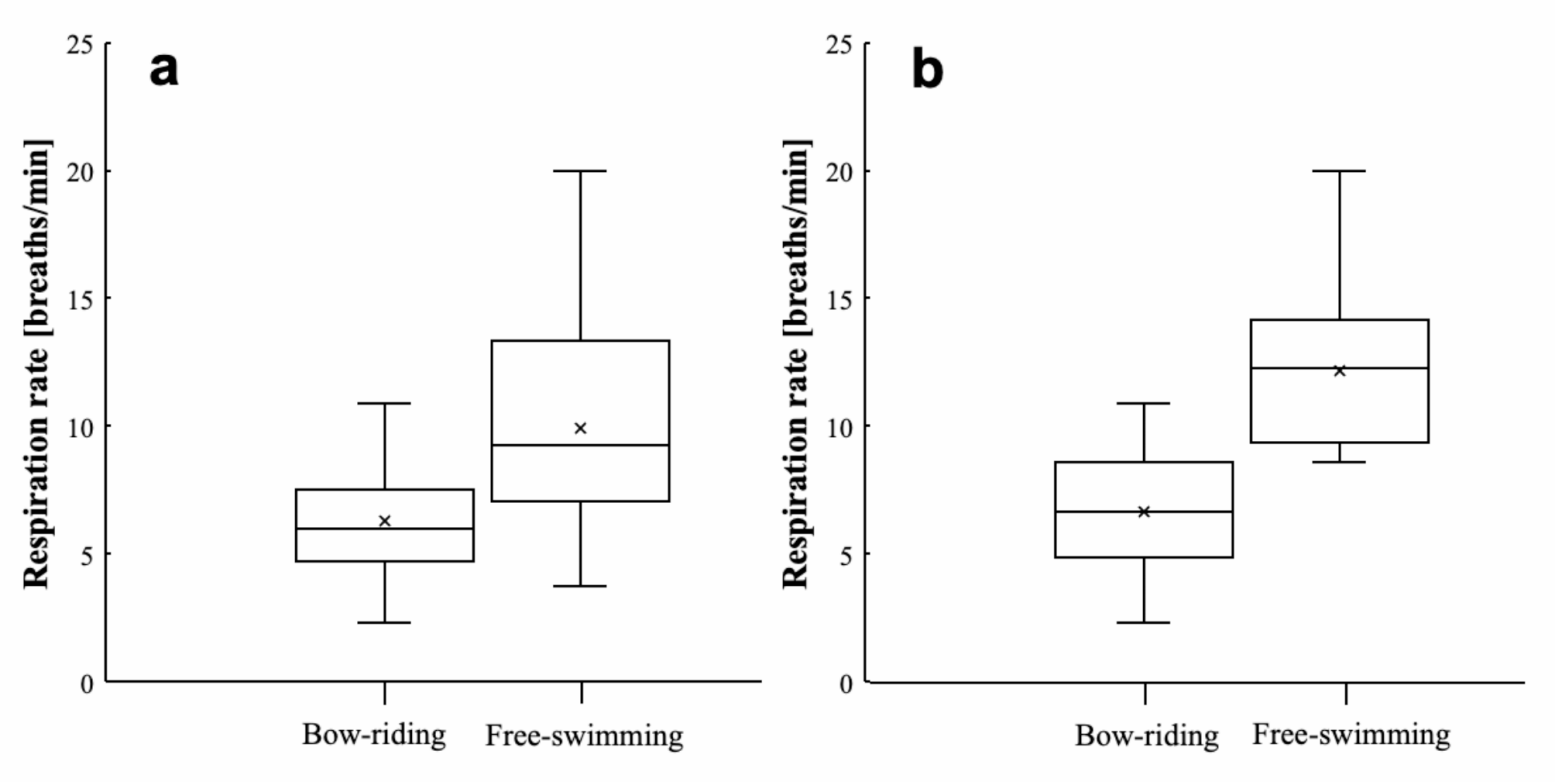
(**a**) Respiration rates of bow-riding (*n* = 27) and free-swimming (*n* = 27) dusky dolphins (*Lagenorhynchus obscurus*) travelling at the same speeds (2.2–6.6 m/s); Mean respiration rate was significantly lower for bow-riding dolphins (*p* < 0.001). (**b**) Respiration rates of bow-riding (*n* = 17) and free-swimming (*n* = 17) dusky dolphins (*Lagenorhynchus obscurus*) travelling at speeds > 4.0 m/s (4.3–6.6 m/s); Mean respiration rate was significantly lower for bow-riding dolphins (*p* < 0.001). Boxes represent first and third quartiles, solid horizontal lines inside boxes represent medians, crosses represent the mean, whiskers above and below boxes show 10th and 90th percentiles.

**Fig. 3:**
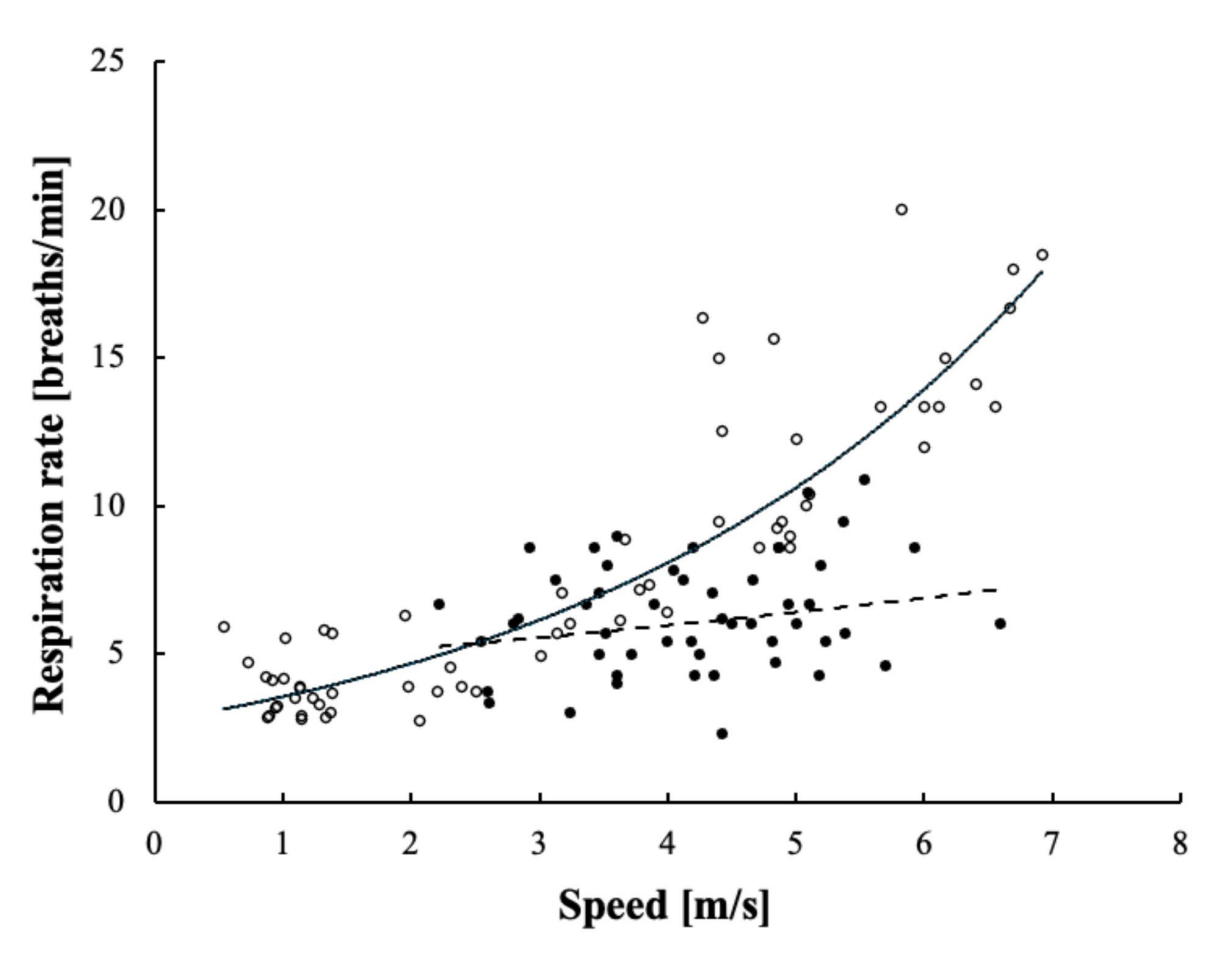
Respiration rate (breaths/min) as a function of swimming speed (0.9–6.9 m/s) for dusky dolphins (*Lagenorhynchus obscurus*). Black and white circles represent bow-riding (*n* = 62) and free-swimming (*n* = 51) dolphins, respectively. The dashed line represents the equation y = 4,48e^0,0716x^, which best describes the relationship between respiration rate and speed for bow-riding dolphins (R^2^ = 0.05). The solid black line represents the equation y = 2.71e^0.279x^, which best describes the relationship between respiration rate and speed for free-swimming dolphins (R^2^ = 0.82).

Bow-riding (*n* = 27) and free-swimming (*n* = 27) dolphins travelling at the same speed were compared (Fig. 2a). Video segments in which dolphins travelled in a linear direction but at a different speed than the other swimming context were excluded from statistical analysis (bow-riding *n* = 24; free-swimming *n* = 35). No violations from normality were detected for swimming speed or respiration rate. Deviations from homoscedasticity were found for respiration rate and corrected through use of the Welch’s t-test. Based on the potential error calculated from the UAS GPS accuracy, speed differences up to 1.5% were tolerated. No significant difference was detected for the two speed distributions (*t* = -0.09, df = 52, *p* = 0.929). Respiration rates ranged from 2.3 to 20.0 breaths/min (bow-riding mean ± SE = 6.3 ± 0.4; free-swimming mean ± SE = 9.9 ± 0.8). The respiration rate of bow-riding dolphins was not statistically influenced by speed (R^2^ = 0.05) (Fig. 3). The respiration rate of free-swimming dolphins increased with swimming speed following an exponential relationship best described by the equation y = 2.71e^0.279x^ (R^2^ = 0.82, = 62) (Fig. 3). The mean (± SE) respiration rate of bow-riding dolphins (6.3 ± 0.4) was significantly lower than free-swimming dolphins (9.9 ± 0.8; *t* = -4.14, df = 39, *p* < 0.001). The greatest differences between respiration rates were observed for dolphins swimming > 4.0 m/s (bow-riding mean ± SE = 6.7 ± 0.5; free-swimming mean ± SE = 12.1 ± 0.8) (Fig. 2b).

**Fig. 4:**
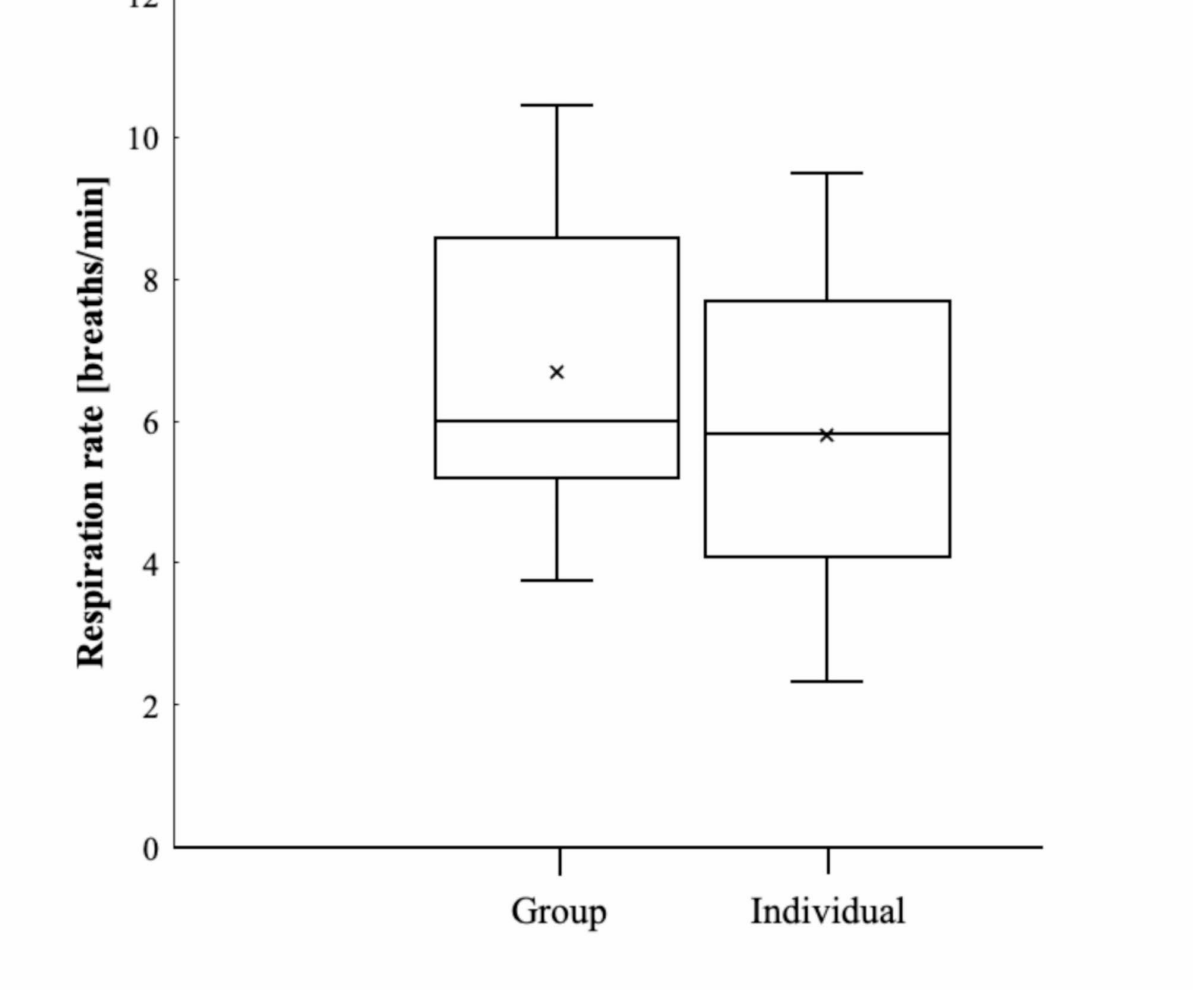
Respiration rates of dusky dolphins (*Lagenorhynchus obscurus*) bow-riding in group (*n* = 12; 2 to 4 individuals simultaneously) and individually (*n* = 12) travelling at the same speeds (2.6–5.4 m/s); Mean respiration rate did not differ significantly between the two contests (*p* = 0.315). Boxes represent first and third quartiles, solid horizontal lines inside boxes represent medians, crosses represent the mean, whiskers above and below boxes show 10th and 90th percentiles.

The mean respiration rates for dolphins bow-riding individually (*n* = 12) and in groups with two to four conspecifics (*n* = 12) travelling at the same speed were compared (Fig. 4). No violations from normality and homoscedasticity were detected for swimming speed nor respiration rate. The mean (± SE) respiration rates of dolphins bow-riding individually (5.8 ± 0.6) did not differ significantly from dolphins bow-riding with conspecifics (6.7 ± 0.6; *t* = 1.03, df = 22, *p* = 0.315).

## Discussion

Bow-riding dolphins had lower respiration rates than free-swimming dolphins travelling at the same speeds. The average 36% reduction in respiration rate of bow-riding dusky dolphins is remarkably similar to the 37% reduction in respiration rate of wake-riding compared to free-swimming bottlenose dolphins^2^. Although bow- and wake-riding present different hydrodynamic challenges for dolphins^6^, the energetic advantages of swimming in the pressure waves created by advancing vessels may be consistent.

The respiration rates of bow-riding compared to free-swimming dusky dolphins almost halved when travelling at speeds exceeding 4 m/s (45% average reduction). Free-swimming dusky dolphins transitioned from submerged swimming to porpoising (i.e., alternations between near-surface swimming and breaching) at the speed of 4.4 m/s^15^. Dusky dolphins in the bow-riding position were not observed porpoising at any speed; bow-riding dusky dolphins may save energy at speeds typically associated with porpoising by remaining submerged and not breaching^30^. Bow-riding dolphins may also require less surface time during respirations and limit the energy expenditure associated with surface drag. Bow-riding common dolphins (*Delphinus delphis*) and pantropical spotted dolphins (*Stenella attenuata*) significantly reduced the total time of blowhole exposure to air compared to free-swimming dolphins travelling at the same speed^31^. The locomotion advantage of bow-riding appears to be influenced by vessel speed with the greatest reduction of dusky dolphin respiration rate occurring at speeds > 4 m/s, which corresponds to the speed at which the boat transitioned to be on a plane. Bow-riding dusky dolphins showed no interest in bow-riding at vessel speeds < 2 m/s, perhaps because the lift generated by the bow may not provide a sufficient physiological advantage. The maximum speed while bow-riding was 6.6 m/s; dolphins did not engage or quickly left the bow-riding position when the research vessel travelled faster. The maximum speed for free-swimming dusky dolphins recorded in this study was 6.9 m/s, which is lower than the previous calculation of 10 m/s^32^. While different techniques can bias speed measurements^33^, more data on fast travelling dusky dolphins are necessary to establish maximum swimming speed.

Factors such as hull shape (e.g., blunt-prowed vs. sharp-prowed) and vessel mass can determine the lift power generated by the bow wave^7,34,35^ and likely the energy saved by bow-riding. Bottlenose dolphins are recurrently observed bow-riding on the pressure waves produced by large and low maneuverable tankers and freighters in the Gulf of Mexico^6^. Bow-riding often occurs when vessels maintain a constant speed and direction^3^. Vessels advancing with frequent changes in speed and erratic maneuvers tend to elicit strong avoidance responses from dolphins^36,37^. However, dolphins that frequently engage in bow-riding can suffer long-term consequences as they spend less time foraging and resting, especially in areas with intense vessel traffic and/or high levels of dolphin-watching activities^12,13^. Limiting vessel speeds below 2 m/s in proximity of dolphins may reduce bow-riding occurrence and its potentially detrimental effects, which is recommended for recreational boaters with dolphin in close proximity.

By establishing the relationship between respiration rate and swimming speed for a cetacean species, it is possible to quantify the energy requirements associated with horizontal avoidance responses characterized by swimming speed increases^38–40^. Such quantification can augment understanding the physiological consequences of anthropogenic disturbances to cetaceans^14,41^. The use of a UAS as a non-invasive tool to characterize the relationship between respiration rate and swimming speed was successfully tested in this study and can be applied to other cetacean species. The respiration advantage provided by the echelon position^8^, and the disadvantage associated with events that cause mother-calf separation^42^, can be also quantified using the respiration rate as a proxy of energy expenditure. Other proxies such as tail stroke frequency can be considered, although detecting fluke movements in the UAS footage is not consistently feasible^9^. Factors such as water turbidity, glare, weather and light conditions, and dorsal pigmentation of the focal dolphin affect the detectability of behaviors occurring underwater^43^. No behavioral reactions to the UAS were observed at the flight altitude to 20 m, although lower altitudes may elicit dolphin behavioral and/or physiological responses^20,44,45^.

Anatomical and physiological differences in various cetacean species and vessel type are likely to affect the locomotion advantage of bow-riding. The bow size and shape limit the maximum number of dolphins benefitting from the pressure wave lift; competition commonly occurs among dolphins for the optimal position in front of the bow^6,7^. Bow-riding is generally a group behavior and may co-occur with vocalizations associated with socializing contexts^46^. We found no significant difference in the mean respiration rates of dusky dolphins bow-riding individually and with conspecifics. Although we did not find a locomotion advantage to swimming in a conspecific’s draft as observed in killer whales^9^, we recommend that future studies investigate bow-riding dolphins’ positions and distances from each other and assess if multiple dolphins swimming in the bow and wake of the same vessel simultaneously experience matching energy savings. We did not measure the distance between a dolphin and a bow wave as dolphin positions varied throughout focal follows (although always within one body length of the boat). Future studies may use modelling approaches to explore how proximity to and position within a pressure wave alter dolphin swimming energetics as there are different pressure fields within large surface waves^34^. Future research is warranted to explore if one type of hull-produced pressure wave saves dolphins more energy, and if the patterns are widespread across more small oceanic dolphin species and geographic locations. While most species of oceanic dolphins have been reported bow-riding, anthropogenic interactions in different geographical areas seem to influence the occurrence^6^. Our study indicates that bow-riding is an efficient way for dusky dolphins to save energy when travelling. Non-mutually exclusive functional hypotheses such as socializing and “fun” require investigation.

## Data Availability

The experimental data and the simulation results that support the findings of this study are available in Figshare with the identifier: 10.6084/m9.figshare.25951675. Contact the main author at lorenzo.fiori@tamucc.edu should you be interested in requesting the data from this study.
